# Antibacterial Effects of the iVac Endodontic Irrigation System Within Dentinal Tubules: An In Vitro Confocal Microscopy Study

**DOI:** 10.3390/jfb17100524

**Published:** 2026-10-17

**Authors:** Narcisa Mandras, Damiano Pasqualini, Gianmarco Baima, Vittorio Fenoglio, Janira Roana, Mishel Londo, Nicola Scotti, Mario Alovisi

**Affiliations:** 1 Department of Sciences of Public Health and Pediatrics, University of Turin, 10126 Turin, Italy; narcisa.mandras@unito.it (N.M.); janira.roana@unito.it (J.R.); 2 Department of Surgical Sciences, C.I.R. Dental School, University of Turin, 10126 Turin, Italy; damiano.pasqualini@unito.it (D.P.); gianmarco.baima@icloud.com (G.B.); fenoglio.vittorio@gmail.com (V.F.); mishel_999@hotmail.com (M.L.); nicola.scotti@unito.it (N.S.)

**Keywords:** apical periodontitis, endodontic irrigation, negative pressure, ultrasonic irrigation, confocal laser scanning microscopy

## Abstract

**Background:** This study compared the antibacterial efficacy of iVac and conventional syringe-and-needle irrigation, used with an identical standardized antimicrobial irrigant regimen, by evaluating bacterial viability and the depth of membrane-compromised bacterial cells within dentinal tubules. **Methods:** Forty-two extracted human teeth were instrumented, sterilized, and inoculated with *Enterococcus faecalis* ATCC 29212. Specimens were randomly assigned to iVac irrigation (*n* = 20) or conventional 30G syringe-and-needle irrigation (*n* = 20). Both protocols employed the same irrigation sequence consisting of 5% NaOCl, 10% EDTA, 5% NaOCl, and saline. After treatment, the teeth were sectioned, stained with the BacLight viability kit, and analyzed by confocal laser scanning microscopy (CLSM) to determine the mean proportion of red staining (MPRS) and mean penetration depth (MPD) defined as the depth within dentinal tubules at which PI-associated red fluorescence corresponding to membrane-compromised bacterial cells was detected in the coronal, middle, and apical thirds. Statistical significance was set at *p* < 0.05. **Results:** No significant difference in mean proportion of red staining (MPRS) was found between the two groups (*p* = 0.24). In contrast, mean penetration depth (MPD) was significantly greater with iVac overall (*p* = 0.021), particularly in the coronal and middle thirds (*p* = 0.015 and *p* = 0.032, respectively). No significant difference was observed in the apical third (*p* = 0.12). **Conclusions:** Under the present experimental conditions, the iVac and conventional syringe-and-needle irrigation protocols resulted in comparable overall bacterial viability outcomes. However, the iVac protocol was associated with a significantly greater depth of membrane-compromised bacterial cells within dentinal tubules, particularly in the coronal and middle thirds.

## 1. Introduction

Apical periodontitis (AP) is an inflammatory disease of the periapical tissues caused by a well-organized polymicrobial biofilm established within the root canal system, which plays a pivotal role in disease pathogenesis. Given that AP affects nearly half of the global population, it represents a significant public health burden and a major challenge for contemporary dental care [1,2,3].

Biofilms consist of structured microbial communities embedded within a self-produced extracellular polymeric matrix adherent to moist surfaces [4,5]. Compared with planktonic microorganisms, biofilm-associated bacteria exhibit enhanced resistance to antimicrobial agents and host defenses, making complete eradication particularly challenging. Although nonsurgical root canal treatment is a highly predictable procedure with favorable long-term outcomes [6,7], persistent intra-radicular infection remains the leading cause of post-treatment apical periodontitis and endodontic failure [5,8,9,10,11,12].

Mechanical instrumentation combined with chemical irrigation substantially reduces the intracanal microbial load but cannot completely eliminate microorganisms because of the anatomical complexity of the root canal system. Isthmuses, accessory canals, fins, and other irregularities frequently remain untouched by endodontic instruments, with more than one-third of the canal surface reportedly left uninstrumented [11]. Consequently, viable bacteria may persist within these inaccessible areas and dentinal tubules, particularly in the apical third, compromising treatment outcomes [8,9,10,11,12,13,14,15].

Instrumentation also generates a smear layer composed of organic and inorganic debris that covers dentinal walls and occludes dentinal tubules, thereby limiting the penetration of irrigants and intracanal medicaments [16,17,18]. Moreover, accumulated dentine debris may reduce the tissue-dissolving efficacy of sodium hypochlorite (NaOCl) and contribute to the formation of the apical vapor lock effect, further impairing irrigant exchange in the apical region [19,20]. Therefore, effective irrigation is essential to enhance canal debridement beyond the mechanical action of instruments [21].

Conventional syringe-and-needle irrigation remains the most widely used irrigation technique. However, its effectiveness is highly dependent on needle insertion depth, as irrigant replacement is limited to approximately 1–1.5 mm beyond the needle tip [22,23]. In narrow or curved canals, safe placement of the needle close to working length is often unfeasible, reducing irrigant exchange and potentially allowing bacterial persistence. Furthermore, conventional needle irrigation carries a greater risk of apical extrusion of NaOCl than alternative irrigation techniques [24,25].

To overcome these limitations, several irrigation strategies have been developed to improve irrigant delivery and activation [26]. Passive ultrasonic irrigation (PUI) enhances irrigant effectiveness through acoustic streaming and cavitation, thereby improving debris removal and disinfection [27,28,29,30,31]. Alternatively, apical negative-pressure systems deliver irrigants coronally while simultaneously aspirating them at working length, promoting continuous irrigant exchange and reducing the risk of apical extrusion [25,29,30]. Despite the promising results reported for both approaches, limited evidence is available regarding systems combining ultrasonic activation with apical negative pressure [32].

The iVac endodontic irrigation system (Pac-Dent, Brea, CA, USA) was developed to integrate these two principles into a single device. The system incorporates a polyether ether ketone (PEEK) microcannula connected to an ultrasonic handpiece and an aspiration unit, allowing simultaneous irrigant activation, delivery, and suction [33]. This combined mechanism may enhance irrigant penetration and antimicrobial efficacy while minimizing apical extrusion.

Therefore, the aim of the present in vitro study was to compare two root canal irrigation protocols—iVac and conventional syringe-and-needle irrigation—using an identical standardized antimicrobial irrigant regimen, by evaluating bacterial viability and the depth within dentinal tubules at which membrane-compromised bacterial cells were detected through confocal laser scanning microscopy. The null hypothesis was that no significant differences would be observed between the two irrigation protocols in terms of bacterial viability or the depth of membrane-compromised bacterial cells within dentinal tubules.

## 2. Materials and Methods

### 2.1. Study Design and Specimen Preparation

This in vitro study was conducted in accordance with the PRILE 2021 guidelines for reporting laboratory studies in Endodontology [34] and was approved by the Local Ethics Committee of the University of Turin (C.I.R. Dental School, Turin, Italy). An a priori sample size calculation was performed using G*Power version 3.1.4 (Kiel University, Kiel, Germany), with an α error probability of 0.05 and a statistical power of 80%. The analysis indicated that a minimum of 20 specimens per group was required.

Forty-two extracted human single-rooted teeth with mature apices were selected. Following extraction, residual soft tissues and debris were removed from the root surfaces using Gracey curettes. The specimens were then immersed in a 0.01% sodium hypochlorite (NaOCl) solution at 4 °C for 24 h (Niclor 5, OGNA, Muggiò, Italy) and subsequently stored in saline solution at 4 °C until experimental use, which occurred within 1 month of extraction. Teeth presenting caries, restorations, root fractures, previous endodontic treatment, accessory canals, or discoloration were excluded. Following access cavity preparation, working length was established using a size 10 K-file under an operating microscope. Root canals were instrumented with the ProTaper Next system (X1–X3) after glide-path preparation with ProGlider (Dentsply Sirona, Ballaigues, Switzerland). Apical patency was maintained with a size 10 K-file throughout instrumentation. During canal preparation, irrigation was performed using alternating 5% sodium hypochlorite (NaOCl) and 10% EDTA delivered with a 30-G side-vented needle. After instrumentation, specimens were sterilized in an autoclave (121 °C for 20 min), and sterility was confirmed by incubation in Brain Heart Infusion (BHI) broth at 37 °C for 24 h. One specimen served as the negative control.

### 2.2. Bacterial Contamination

*Enterococcus faecalis* ATCC 29212 was cultured overnight in BHI broth, and the bacterial suspension was adjusted to approximately 3 × 10^8^ CFU/mL. Under sterile conditions, the specimens were inoculated with the bacterial suspension and centrifuged to facilitate bacterial penetration into the dentinal tubules. The specimens were subsequently maintained in inoculated BHI broth and incubated under aerobic conditions at 37 °C for 21 days. Fresh BHI broth was replaced every 48 h throughout the incubation period to maintain bacterial viability and nutrient availability. The bacterial suspension was not actively delivered into the root canals using a needle. Following the 21-day contamination period, the specimens were removed from the culture medium and subjected to the assigned irrigation protocols [35].

### 2.3. Irrigation Protocols

Specimens were randomly allocated to two experimental groups (*n* = 20 each). In the iVac group (*n* = 20), irrigation was performed using the iVac system (Pac-Dent, Brea, CA, USA), consisting of a PEEK microcannula connected to a piezoelectric ultrasonic unit and an aspiration line generating apical negative pressure. The microcannula was positioned 1 mm short of the working length and activated at 70% power according to the manufacturer’s recommendations, avoiding contact with the canal walls. In the needle irrigation group (*n* = 20), irrigants were delivered using a conventional 30-G side-vented needle (Kerr Endodontics, Brea, CA, USA). Both protocols employed the same irrigation sequence: 5% NaOCl (60 s), 10% EDTA (60 s), 5% NaOCl (60 s), and saline (60 s). Irrigants were delivered at a constant flow rate (0.1 mL/s), and identical solution volumes were used in both groups. Following irrigation, canals were dried with sterile paper points. All procedures were performed by a single experienced endodontist.

### 2.4. Specimens Sectioning

Following treatment, crowns were removed at the cementoenamel junction (CEJ) to obtain a standardized root length of 12 mm. Roots were sectioned perpendicular to the long axis using a precision diamond saw under continuous water cooling, producing 1-mm-thick dentin slices. After sectioning, the slices were thoroughly rinsed with saline to remove loose cutting debris. The surfaces intended for CLSM examination were then superficially polished to remove sectioning-related surface debris and expose a fresh dentinal surface, followed by a further rinse with saline before confocal imaging. All specimens underwent the same standardized sectioning, polishing, and rinsing procedure. Specimens were categorized as coronal (0–4 mm), middle (4–8 mm), or apical (8–12 mm) according to their anatomical location [36].

### 2.5. CLSM Analysis

Specimens were stained using the LIVE/DEAD BacLight bacterial viability kit (Invitrogen, Eugene, OR, USA), containing SYTO 9 and propidium iodide (PI), to distinguish viable from membrane-compromised bacterial cells [37]. After staining, slices were rinsed to expose a fresh dentinal surface before examination. Confocal laser scanning microscopy (Leica TCS-SPE; Leica Microsystems, Mannheim, Germany) was performed using standardized acquisition parameters. Images were collected with ×40 and ×63 oil-immersion objectives using sequential scanning to prevent fluorescence overlap. Measurements were performed in the coronal, middle, and apical thirds by blinded examiners. For each specimen, two outcome measures were recorded:

- Mean Proportion of Red Staining (MPRS): percentage of red fluorescence relative to total fluorescence, used as an indicator of the proportion of bacterial cells with compromised membrane integrity.
- Mean Penetration Depth (MPD): mean depth (μm) at which red fluorescence associated with propidium iodide-stained, membrane-compromised bacterial cells was detected within dentinal tubules, representing the depth of the antibacterial effect within the dentinal tubules rather than a direct measurement of irrigant penetration.

### 2.6. Statistical Analysis

Descriptive statistics were calculated for MPRS and MPD. Inferential statistical analyses were performed to compare MPRS and MPD between the two irrigation protocols. MPD was also compared between the two irrigation protocols separately for the coronal, middle, and apical thirds. For root-level analyses, observations were stratified according to anatomical region and irrigation group. Coronal observations were compared between the iVac and conventional irrigation groups, and the same separate between-group comparisons were performed for the middle and apical regions. Comparisons among different root levels within the same tooth were not performed. Tooth identity was not included as a clustering variable in the original statistical analysis. Statistical significance was set at *p* < 0.05.

## 3. Results

**Figure 1:**
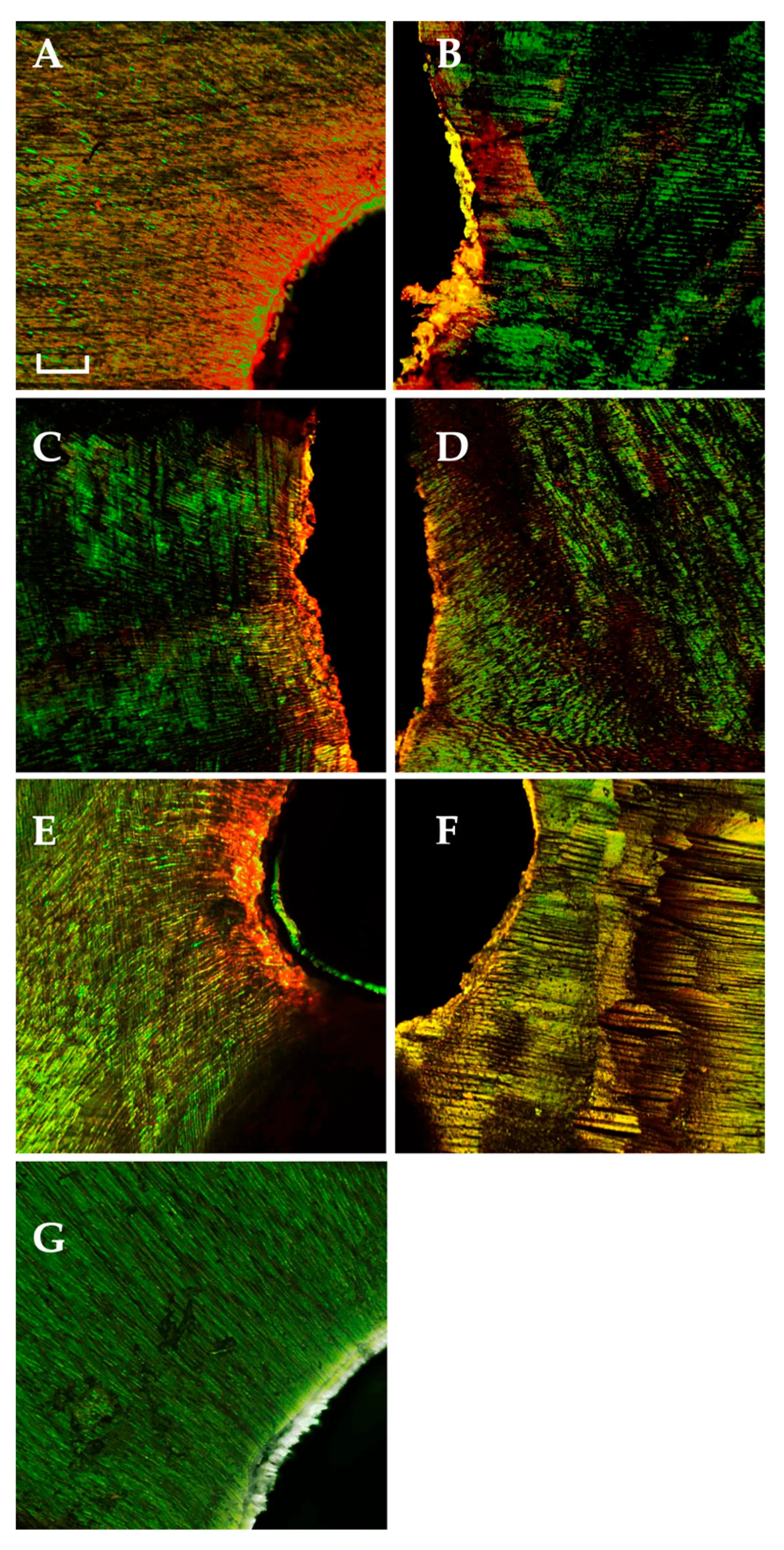
Representative confocal laser scanning microscopy (CLSM) images showing the depth of red fluorescence associated with membrane-compromised bacterial cells within dentinal tubules following conventional syringe-and-needle irrigation and iVac irrigation. (**A**) iVac, coronal third; (**B**) Conventional irrigation, coronal third; (**C**) iVac, middle third; (**D**) conventional irrigation, middle third; (**E**) iVac, apical third; (**F**) conventional irrigation, apical third; (**G**) representative CLSM image of the positive control following the bacterial contamination protocol and before experimental irrigation, showing bacterial fluorescence extending from the root canal lumen into the dentinal tubules. Green fluorescence indicates SYTO 9 staining, whereas red fluorescence indicates propidium iodide staining. Scale bar: 150 μm.

Sterility testing confirmed the absence of bacterial contamination in all specimens before inoculation with *E. faecalis*. No significant difference in MPRS was observed between the iVac and conventional needle irrigation groups (52.36 ± 4.25% vs. 53.54 ± 3.31%, respectively; *p* = 0.24) (Table 1). In contrast, the mean depth of membrane-compromised bacterial cells within dentinal tubules (MPD) was significantly greater in the iVac group than in the conventional needle irrigation group (359.82 ± 78.10 μm vs. 299.62 ± 51.85 μm, respectively; *p* = 0.021) (Table 1). When analyzed according to root level, MPD was significantly greater with iVac in the coronal (384.44 ± 89.24 μm vs. 309.23 ± 36.13 μm; *p* = 0.015) and middle thirds (375.78 ± 51.48 μm vs. 290.43 ± 38.12 μm; *p* = 0.032), whereas no significant difference was observed in the apical third (319.26 ± 38.92 μm vs. 299.20 ± 33.10 μm; *p* = 0.12) (Table 2) (Figure 1). These findings indicate that PI-associated red fluorescence corresponding to membrane-compromised bacterial cells was detected at a significantly greater depth within dentinal tubules in the iVac group in the coronal and middle thirds, whereas no significant difference was observed in the apical third.

## 4. Discussion

The present in vitro study compared the antibacterial effects of the iVac irrigation protocol with those achieved using conventional syringe-and-needle irrigation under an identical antimicrobial irrigant regimen. Not statistically significant difference was observed in the overall proportion of membrane-compromised bacterial cells between the two irrigation protocols. In contrast, PI-associated red fluorescence was detected at a significantly greater depth within dentinal tubules in the iVac group overall, with significant differences in the coronal and middle thirds but not in the apical third. Therefore, the null hypothesis was partially rejected. Importantly, this depth parameter should not be interpreted as a direct measurement of irrigant penetration, because no fluorescent tracer was incorporated into the irrigating solutions. Rather, it indicates the maximum depth at which membrane-compromised bacterial cells were detected under the present experimental conditions.

To date, the available evidence regarding the iVac system is limited. Previous investigations have mainly focused on its ability to improve smear layer removal and debridement of simulated anatomical complexities, reporting encouraging results compared with conventional irrigation protocols [38,39]. To the best of our knowledge, no previous investigation has specifically assessed both bacterial viability and the depth distribution of membrane-compromised bacterial cells following use of the iVac protocol by CLSM in an *E. faecalis* infection model. Our findings are generally consistent with those reported by Gomes et al. [40], who observed comparable antimicrobial efficacy between iVac and conventional irrigation in a multispecies biofilm model.

The anatomical complexity of the root canal system and the presence of uninstrumented areas, smear layer, and accumulated dentinal debris may limit irrigant penetration and chemical disinfection [10,11,16,17,18,19]. Accordingly, irrigant activation strategies have been developed to improve irrigant exchange and penetration into areas that are inaccessible to mechanical instrumentation [26,27,28,29,30,31,32,41,42]. In the present study, PI-associated red fluorescence was detected at greater depths within dentinal tubules following the iVac protocol than following conventional syringe-and-needle irrigation, particularly in the coronal and middle thirds.

The greater depth of PI-associated red fluorescence observed with the iVac protocol may reflect a more extensive antibacterial effect within dentinal tubules. The iVac system incorporates both ultrasonic activation and apical negative pressure, which may influence irrigant exchange and interaction with canal walls. However, because irrigant penetration was not directly measured in the present study and the two components of the iVac system were not evaluated separately, the mechanism underlying the greater depth of membrane-compromised bacterial cells cannot be determined from the present data. Previous studies have shown that ultrasonic activation can enhance irrigant movement and wall shear stress [43,44,45,46]; these fluid-dynamic effects may provide a plausible mechanistic context for the present findings, but this interpretation remains indirect.

Previous studies have also suggested that ultrasonic activation can promote the penetration of endodontic materials into dentinal tubules, supporting the concept that acoustic energy may facilitate fluid transport through dentinal structures [31,32]. These studies provide indirect evidence of a potential role of ultrasonic agitation in improving the penetration of liquid materials into dentinal tubules and may therefore provide a plausible mechanistic context for the present findings. Therefore, although these fluid-dynamic mechanisms may provide a plausible context for the greater depth of PI-associated red fluorescence observed with iVac, the present findings cannot establish whether iVac resulted in greater irrigant penetration into dentinal tubules.

Unlike conventional ultrasonic irrigation, the iVac system combines ultrasonic activation with apical negative pressure. Negative-pressure irrigation systems, such as EndoVac, have been reported to provide effective irrigant exchange at working length while minimizing the risk of apical extrusion [29,30]. By continuously aspirating irrigants from the apical portion of the canal, negative-pressure systems promote the delivery and exchange of fresh solution throughout the canal space while limiting vapor lock formation and reducing hydrostatic pressure beyond the apical foramen. These fluid-dynamic characteristics may be relevant when interpreting the greater depth of PI-associated red fluorescence observed with iVac. However, because apical negative pressure was not evaluated independently and irrigant penetration was not directly measured, the observed difference in MPD cannot be specifically attributed to this component of the iVac system.

Notably, the greater MPD observed with iVac was significant in the coronal and middle thirds but not in the apical third. This finding indicates that membrane-compromised bacterial cells were detected at greater depths within the dentinal tubules in the coronal and middle thirds following the iVac protocol, whereas this difference was not observed apically. The smaller dimensions and greater anatomical constraints of the apical region may have influenced the interaction of the irrigation protocol with this portion of the root canal system and may therefore provide a possible explanation for the absence of a significant difference in MPD at the apical level. However, since irrigant penetration was not directly assessed in the present study, no conclusions can be drawn regarding differences in irrigant penetration along the root length or the specific contribution of ultrasonic activation and apical negative pressure to the observed depth of membrane-compromised bacterial cells. Further studies specifically designed to investigate irrigant fluid dynamics and dentinal penetration, using dedicated methods for the direct assessment of irrigant penetration, are needed to clarify the mechanisms underlying the present findings, particularly at the apical level.

The absence of a significant difference in overall bacterial viability despite the significantly greater MPD of PI-associated red fluorescence observed with iVac deserves consideration. MPRS and MPD describe different aspects of the CLSM findings: MPRS reflects the overall proportion of PI-associated red fluorescence relative to total fluorescence and therefore provides an indicator of the proportion of bacterial cells with compromised membrane integrity, whereas MPD describes the depth within dentinal tubules at which PI-associated red fluorescence was detected. Consequently, detection of membrane-compromised bacterial cells at greater depths does not necessarily imply a greater overall proportion of membrane-compromised cells. Several factors may have contributed to the present findings. First, sodium hypochlorite exerts rapid bactericidal activity once direct contact with microorganisms is achieved, and both irrigation protocols delivered identical irrigant volumes, concentrations, exposure times, and flow rates. Consequently, the antimicrobial effect may have approached a plateau under the present experimental conditions, thereby reducing the likelihood of detecting differences in overall MPRS between the two protocols. At the same time, the fluid-dynamic characteristics of the iVac protocol may provide a plausible context for the greater depth at which PI-associated red fluorescence was detected. However, because irrigant penetration was not directly measured and ultrasonic activation and apical negative pressure were not evaluated independently, the mechanism responsible for the greater MPD cannot be established from the present findings. Finally, bacterial viability was evaluated using a mature *E. faecalis* biofilm, a microorganism commonly used as a model for evaluating endodontic disinfection strategies because of its persistence and resistance to antimicrobial procedures [46]. These biological characteristics may further reduce the likelihood of detecting differences in overall bacterial viability between irrigation protocols.

Persistent endodontic infections are characterized by polymicrobial microbial communities rather than by a single bacterial species, with their composition potentially varying according to the ecological conditions of the root canal system and the stage of infection [1,5,8,9,24]. Interactions among microorganisms within these communities may contribute to biofilm organization, persistence, and tolerance to antimicrobial procedures, making the microbiological conditions of persistent infections substantially more complex than those reproduced by a monospecies laboratory model. Nevertheless, *E. faecalis* remains a widely used microorganism in experimental endodontic infection models because of its ability to persist under adverse environmental conditions and its association with secondary and persistent endodontic infections. In the present study, the use of a monospecies *E. faecalis* biofilm allowed standardization of the experimental conditions and reproducible quantitative assessment of bacterial viability. However, this model cannot reproduce the ecological complexity and interspecies interactions characteristic of clinical endodontic infections. Future studies employing polymicrobial biofilms would therefore better reproduce the microbiological conditions encountered clinically and may provide additional information regarding the performance of novel irrigation systems.

Another clinically relevant aspect concerns irrigant safety. Sodium hypochlorite extrusion beyond the apical foramen, although uncommon, represents one of the most severe complications of root canal irrigation and may result in extensive tissue injury, severe pain, swelling, and ecchymosis. Negative-pressure irrigation systems have been developed specifically to minimize this risk by aspirating irrigants from the apical region rather than forcing them toward the periapical tissues. Although apical extrusion was not assessed in the present study, the negative-pressure mechanism incorporated into the iVac system may offer a potential safety advantage. However, dedicated studies evaluating apical irrigant extrusion are required before conclusions regarding its safety can be drawn.

A major strength of the present study is the strict standardization of the irrigation protocols. Both groups received the same irrigant sequence, concentrations, exposure times, flow rates, and total irrigant volumes, allowing differences to be attributed primarily to the irrigation system rather than to variations in the chemical protocol. Furthermore, the iVac device was operated according to the manufacturer’s recommendations, thereby reproducing its intended clinical use.

This study also presents several limitations. First, although centrifugation and prolonged incubation were used to facilitate bacterial penetration into dentinal tubules, the experimental contamination protocol cannot ensure perfectly uniform bacterial colonization throughout the entire depth and extent of the dentinal tubule system. Nevertheless, all specimens underwent the same standardized contamination procedure before random allocation to the experimental groups. In addition, although standardized sectioning under continuous water cooling, saline rinsing, and superficial polishing were performed to minimize cutting-related surface debris and expose a fresh dentinal surface, potential alterations of the superficial bacterial distribution resulting from specimen preparation cannot be completely excluded. Second, the experimental model employed extracted single-rooted teeth with relatively simple canal anatomy, which does not fully reproduce the anatomical variability encountered clinically. Third, the use of a monospecies *E. faecalis* biofilm cannot replicate the complexity and interspecies interactions characteristic of polymicrobial endodontic infections. Finally, although root-level analyses were stratified by anatomical region to account for the anatomical and structural differences among the coronal, middle, and apical thirds, potential within-tooth correlation among observations from different root levels cannot be completely excluded and should be considered when interpreting the findings. Future investigations should evaluate the performance of the iVac protocol in anatomically complex teeth, using polymicrobial biofilm models and, ultimately, randomized clinical trials to determine the clinical relevance of the greater depth of membrane-compromised bacterial cells observed in vitro.

## 5. Conclusions

Within the limitations of this in vitro study, the iVac and conventional syringe-and-needle irrigation protocols resulted in comparable overall bacterial viability outcomes under identical chemical irrigation conditions. However, PI-associated red fluorescence corresponding to membrane-compromised bacterial cells was detected at significantly greater depths within dentinal tubules following the iVac protocol, particularly in the coronal and middle thirds. Because irrigant penetration was not directly measured and the individual contributions of ultrasonic activation and apical negative pressure were not evaluated separately, the mechanisms underlying this finding cannot be determined from the present study. Further investigations using fluorescently labeled irrigants or other dedicated methods for directly assessing irrigant penetration are warranted, together with studies employing anatomically complex teeth and polymicrobial biofilm models.

## Figures and Tables

**Table 1:** Comparison of mean proportion of red staining (MPRS) and mean penetration depth (MPD) between conventional syringe-and-needle irrigation and iVac irrigation.

	iVac	Conventional Irrigation	*p*-Value
Mean Proportion of Red Staining (MPRS) (%)	52.36 ± 4.25	53.54 ± 3.31	0.24
Mean Penetration Depth (MPD) (μm)	359.82 ± 78.10	299.62 ± 51.85	0.021

Data are presented as mean ± standard deviation. MPRS, mean proportion of red staining; MPD, mean penetration depth, defined as the depth within dentinal tubules at which PI-associated red fluorescence corresponding to membrane-compromised bacterial cells was detected. Statistical significance was set at *p* < 0.05.

**Table 2:** Comparison of mean penetration depth (MPD) between conventional syringe-and-needle irrigation and iVac irrigation according to root level.

	iVac	Conventional Irrigation	*p*-Value
MPD, coronal third (μm)	384.44 ± 89.24	309.23 ± 36.13	0.015
MPD, middle third (μm)	375.78 ± 51.48	290.43 ± 38.12	0.032
MPD, apical third (μm)	319.26 ± 38.92	299.20 ± 33.10	0.12

Data are presented as mean ± standard deviation. Comparison of mean penetration depth (MPD) of PI-associated red fluorescence corresponding to membrane-compromised bacterial cells between conventional syringe-and-needle irrigation and iVac irrigation according to root level.

## Data Availability

The original contributions presented in this study are included in the article. Further inquiries can be directed to the corresponding author.

## Abbreviations

The following abbreviations are used in this manuscript:

AP	apical periodontitis
CEJ	cementoenamel junction
CLSM	confocal laser scanning microscopy
MPRS	mean proportion of red staining
MPD	mean penetration depth
